# The influence of hepatitis B virus on antiviral treatment with interferon and ribavirin in Asian patients with hepatitis C virus/hepatitis B virus coinfection: a meta-analysis

**DOI:** 10.1186/1743-422X-9-186

**Published:** 2012-09-06

**Authors:** Jun-Ying Liu, Yun-Jian Sheng, Huai-Dong Hu, Qing Zhong, Jing Wang, Shi-Wen Tong, Zhi Zhou, Da-Zhi Zhang, Peng Hu, Hong Ren

**Affiliations:** 1 Department of Infectious Diseases, Institute for Viral Hepatitis, Key Laboratory of Molecular Biology for Infectious Diseases, Ministry of Education, Second Affiliated Hospital of Chongqing Medical University, Chongqing, 400010, China

**Keywords:** Coinfection, Hepatitis B, Hepatitis C, Interferon, Ribavirin

## Abstract

**Background:**

Clinical and laboratory studies have indicated that coinfection with hepatitis B virus (HBV) and hepatitis C virus (HCV) can suppress one another, eliciting a dominant disease phenotype. To assess whether HBV can influence the antiviral effect of treatment on HCV, we performed a meta-analysis to comparatively analyze the response to interferon plus ribavirin treatment in patients with HBV/HCV coinfection and HCV mono-infection.

**Methods:**

Published studies in the English-language medical literature that involved cohorts of HBV/HCV coinfection and HCV mono-infection were obtained by searching Medline, Cochrane and Embase databases. Studies that compared the efficacy of treatment with interferon plus ribavirin in HBV/HCV coinfection and HCV mono-infection were assessed. End-of-treatment virological response (ETVR), sustained virological response (SVR), HCV relapse rate, and alanine aminotransferase (ALT) normalization rate were compared between HBV/HCV coinfection and HCV mono-infection patients.

**Results:**

Five trials involving 705 patients were analyzed. At the end of follow-up serum ALT normalization rates in patients with HCV mono-infection were significantly higher than in patients with HBV/HCV coinfection (odds ratio (OR) = 0.56, 95% confidence interval (CI): 0.40–0.80, *P* = 0.001). The ETVR and SVR achieved in HBV/HCV coinfection patients were comparable to those in HCV mono-infection patients (OR = 1.03, 95% CI: 0.37–2.82, *P* = 0.96 and OR = 0.87, 95% CI: 0.62–1.21, *P* = 0.38, respectively). The rate of relapse for HCV or HCV genotype 1 was not significantly different between HBV/HCV coinfection patients and HCV mono-infection patients (OR = 1.55, 95% CI: 0.98–2.47, *P* = 0.06; HCV genotype 1: OR = 2.4, 95% CI: 1.17–4.91, *P* = 0.19).

**Conclusions:**

Treatment with interferon and ribavirin achieves similar ETVR and SVR in HBV/HCV coinfection and HCV mono-infection. HBV/HCV coinfection patients had distinctively lower end of follow-up serum ALT normalization.

## Background

Hepatitis B virus (HBV) and hepatitis C virus (HCV) are significant human pathogens of global concern. Infection with either hepatitis virus can lead to chronic liver disease, severely degrading quality of life and eventually causing death. The infection elicits a persistent host inflammatory response, which is accompanied by activation of biomolecule signaling cascades that can promote chronic hepatitis, liver cirrhosis, and/or hepatocellular carcinoma (HCC) [1-3].

These two hepatotropic viruses share the same modes of transmission and often appear as a coinfection in geographic locales where HBV or HCV is considered endemic [4-6]. In Taiwan, 11% of patients with HBV–related chronic liver disease also presented with positive immunoreactivity for anti-HCV antibody [5,7]; likewise, 12% of the Taiwanese anti-HCV positive patients had detectable levels of hepatitis B surface antigen (HBsAg) in their serum [4]. In Italy, up to 40% of patients with chronic hepatitis B (CHB) have been reported as anti-HCV positive [8]. Moreover, occult HBV infection has been described in patients with chronic hepatitis C (CHC) [9,10].

Several clinical and laboratory studies have investigated the potential interactions of HBV and HCV and their concomitant affects on the immune response; these studies have revealed that HBV and HCV are capable of suppressing replication of one other in coinfection conditions [11-14]. Clinically, patients with HBV/HCV coinfection have a much higher risk of developing cirrhosis, hepatic failure, and HCC than patients with HBV or HCV mono-infection [14-19].

The current gold standard treatment of CHC is a combination pharmacotherapy using pegylated (PEG) interferon (IFN) and ribavirin [20-22]. Interferon has also been shown as an effective treatment for CHB [23,24]; however, interferon alone is not effective in clearing HCV from patients with HBV/HCV coinfection [25-29]. The combination treatment strategy of interferon plus ribavirin is more effective for HCV clearance in patients with HBV/HCV coinfection, and current treatment guidelines recommend this approach. However, the efficacy of combination treatment in HBV/HCV coinfection has yet to be definitively evidenced as equal to that in HCV mono-infection [30-32]. To this end, we performed a comparative analysis of the therapeutic efficacy of interferon plus ribavirin reported from cohort studies of patients with HBV/HCV coinfection and HCV mono-infection by undertaking a meta-analysis according to PRISMA (Transparent Reporting of Systematic Review and Meta-analyses) criteria [33] (Additional file 1).

## Methods

### Literature search

Relevant studies were identified by searching the Medline, Embase, and Cochrane databases, using the following medical subject headings: “hepatitis B virus and hepatitis C virus coinfection”, “hepatitis B virus and hepatitis C virus dual infection”, “interferon and ribavirin”, and “antiviral therapy”. The scope of the search was restricted to “human” and “English”. We included all cohort studies. The search was carried out in March 2011, without a lower date limit for the search results. The reference lists of all retrieved review articles were manually searched to identify potentially relevant articles missed by the computer search. We used only previously published data, so approval from the ethics committee was not required.

### Inclusion and exclusion criteria

Inclusion criteria for the meta-analysis were as the follows: (1) cohort study design (each group sample size >10); (2) study including an HBV/HCV coinfection group and an HCV mono-infection group; and (3) patients of two groups treated with interferon plus ribavirin. Patient populations were excluded if they featured: (1) coinfection with hepatitis A, D, or E virus, or human immunodeficiency virus (HIV); (2) diagnosis of autoimmune hepatitis, primary biliary cirrhosis, Wilson^’^s disease, decompensated cirrhosis, or overt hepatic failure; (3) clinical evidence of HCC; (4) current or past history of alcohol abuse (alcohol intake ≥20 g daily); or (5) use of antiviral drugs or immunomodulatory drugs at any time within the preceding six months. Any dataset for which sufficient analytic information was not available was also excluded from the meta-analysis.

### Efficacy measures

The primary efficacy end-point was sustained virological response (SVR), which was defined as the proportion of patients with undetectable serum HCV-RNA for at least 24 weeks after treatment. Secondary end points were: end-of-treatment virological response (ETVR), defined as the proportion of patients with undetectable serum HCV-RNA at the end of treatment; biochemical response, defined as normalization of serum alanine aminotransferase (ALT); viral relapse, defined as the proportion of patients with undetectable serum HCV-RNA at the end of treatment but with detectable serum HCV-RNA at follow-up; and viral interaction of HBV and HCV in HCV/HBV coinfection patients.

### Data extraction

Two authors (Liu and Sheng) independently evaluated the retrieved studies according to the inclusion criteria and performed data extraction. The following data were extracted from each paper: (1) number of patients in the study; (2) details of the study design; (3) patient characteristics; (4) treatment doses and duration; and (5) outcome measures, as defined above. Disagreements were resolved by consensus.

### Study quality

Quality of each study was independently assessed by the same two authors (Liu and Sheng) according to the following high-quality features: (1) cohort studies designed with case characteristics (clinical and/or demographic) matched to controls; and (2) presence of a definitive listing of inclusion and exclusion criteria for patients, along with clear definitions of treatment response. When discrepancies arose, a third party (Peng Hu) was consulted.

### Statistical analysis

Meta-analysis was performed using Review Manager Software 5.0 (Cochrane Collaboration, Oxford, United Kingdom), according to recommendations of the manufacturer and the Quality of Reporting of Meta-analyses (QUORUM) guidelines (DerSimonian R, Laird N, et al. 1986; Moher D, Cook DJ, et al. 2000). Statistical analysis for dichotomous variables was carried out. Outcomes were expressed as relative risks (RR) with 95% confidence intervals (CI). If the value 1 was included in the 95% CI, the point estimate of the RR was considered to have reached statistical significance (p < 0.05). The I-squared (I^2^) statistic was used to measure the extent of inconsistency among the results. Heterogeneity was detected using the chi-square (*X*^2^) test. Since the *X*^2^ test lacks power with few studies, we considered significant heterogeneity having been met when both the *X*^2^ value was within the 10% level of significance (*P* < 0.10) and the I^2^ value exceeded 56%. In cases where significant heterogeneity existed, the random effect model was used for analysis, otherwise the fixed effect model was used.

## Results

### Search results and study characteristics

**Figure 1:**
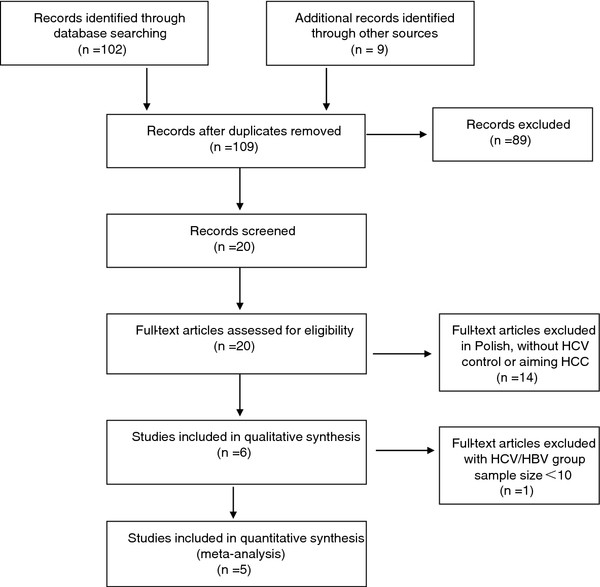
Flow diagram of the meta-analysis study selection process.

A total of 111 studies were identified and screened for retrieval by using the strategy described above. After screening the title or abstract, 91 studies were excluded and 20 were retrieved and subjected to detailed evaluation. By adhering to the inclusion criteria, 15 of those studies were excluded. Finally, five cohort studies [30,32,34-36] were chosen for inclusion in the meta-analysis, which comprised a total of 705 patients (Figure 1).

The basic characteristics of each of the five studies are listed in Table 1. The overall population ethnicity was Asian for all five studies, with one study [34] composed of Chinese and four [30,32,34,36] composed of Taiwanese. Patients entered the cohorts from August 2000 to April 2007. The population size for each of the studies ranged from 54 to 321. The mean age ranged from 44.9 [35] to 51.1 [32] years old. The percentage of males ranged from 61.5% [32] to 75.0% [34]. The mean HCV-RNA level varied from 2.16 log_10_[35] to 6.3 log_10_[35] copies/mL. Patients in three of the studies [30,34,36] were treated with conventional interferon and ribavirin, patients in the other two studies [32,35] were treated with PEG-IFN (having a longer half-life) and ribavirin. Outcomes of the clinical trials included in the meta-analysis are presented in Table 2.

**Table 1:** Characteristics of the clinical trials included in the meta-analysis

**Study**	**Geographic locale**	**Study design**	**Sample size(female/male)**	**Age in years (mean ± SD)**	**HCV-RNA, copies/mL (mean ± SD)**	**HCV genotype 1/non-genotype 1**	**HBV-DNA, copies/mL (mea ± SD)**	**ALT, U/L (mean ± SD)**	**IFN plus ribavirin regimen**		
									**Drugs**	**Course, weeks**	**Follow-up, weeks**
Yu et al. [35]	China	Cohort	C: 10/40	44.9 ± 9.6	5.9 ± 1.4log10	C: 30/20	4.6 ± 0.9log10	97 ± 19	PegIFN-α2a, ribavirin	genotype 1: 48 non-genotype 1: 24	24
			M: 9/37	46.4 ± 11.3	6.3 ± 1.3log10	M: 25/21		93 ± 18			
Liu et al. [32]	Taiwan	Cohort	C: 56/105	51.1 ± 10.3	2.16 ± 3.4 × 10^6^	C: 97/64	UD-4.22 × 10^5^	121.0 ± 85.0	PegIFN-α2a, ribavirin	genotype 1: 48 non-genotype 1: 24	24
			M: 71/89	48.9 ± 10.5	2.29 ± 4.1 × 10^5^	M: 110/50		126.1 ± 81.8			
Chuang et al. [34]	Taiwan	Cohort	C: 11/31	45.04 ± 10.7	5.38 ± 0.96log10	C: 21/21	2.64 ± 2.26log10	92.1 ± 73.0	IFN-α2b	24	24
			M: 22/62	44.9 ± 11.2	5.60 ± 0.94log10	M: 48/36		141.1 ± 105.5			
Hung et al. [30]	Taiwan	Cohort	C: 14/22	48.8 ± 12.6	5.80 ± 1.10og10	C: 17/19	NR	125.8 ± 89.3	IFN-α2b	24	24
			M: 28/44	48.3 ± 12.0	5.80 ± 1.10og10	M: 34/38		164.2 ± 139.9			
Liu et al. [36]	Taiwan	Cohort	C: 5/19	46.5 ± 13.2	2.4 ± 3.1 × 10^6^	C: 14/7	1.3 ± 2.5 × 10^3^	137.0 ± 81.0	IFN-α2a	24	24
			M: 12/18	48.5 ± 9.4	1.9 ± 2.2 × 10^6^	M: 17/13		177.0 ± 192.0			

*C* HBV and HCV coinfection, *M* HCV mono-infection, *NR* not reported, *UD* not detectable by the real-time PCR assay.

**Table 2:** Outcomes of the clinical trials included in the meta-analysis

**Study**	**Sample size (*n*)**	**HCV genotype (*n*)**		**End of treatment**				**End of follow-up**						
				**HCV ETVR (*n*)**			**ALT normalization (*n*)**	**HCV SVR (*n*)**			**HCV relapse (*n*)**			**ALT normalization (*n*)**
		**Genotype 1**	**Non- genotype 1**	**Overall**	**Genotype 1**	**Non-genotype 1**		**Overall**	**Genotype 1**	**Non-genotype 1**	**Overall**	**Genotype 1**	**Non-genotype 1**	
Yu et al. [35]	C: 50	30	20	46	27	19	NR	27	12	15	19	15	4	NR
	M: 46	25	21	32	14	18	NR	26	11	15	6	3	3	NR
Liu et al. [32]	C: 161	97	64	138	82	56	NR	123	70	53	15	12	3	103
	M: 160	110	50	141	95	46	NR	127	85	42	14	10	4	122
Chuang et al. [34]	C: 42	21	21	NR	NR	NR	NR	29	10	19	NR	NR	NR	23
	M: 84	48	36	NR	NR	NR	NR	54	39	15	NR	NR	NR	51
Hung et al. [30]	C: 36	17	19	33	NR	NR	24	25	10	15	8	NR	NR	20
	M: 72	34	38	68	NR	NR	66	51	18	33	17	NR	NR	52
Liu et al. [36]	C: 24	14	7	16	10	6	12	9	3	6	7	NR	NR	9
	M: 30	17	13	25	NR	NR	23	18	NR	NR	5	NR	NR	18

*C* HBV and HCV coinfection, *M* HCV mono-infection, *NR* not reported.

### Comparison of serum ALT normalization rates achieved in HBV/HCV coinfection patients and HCV mono-infection patients

**Figure 2:**
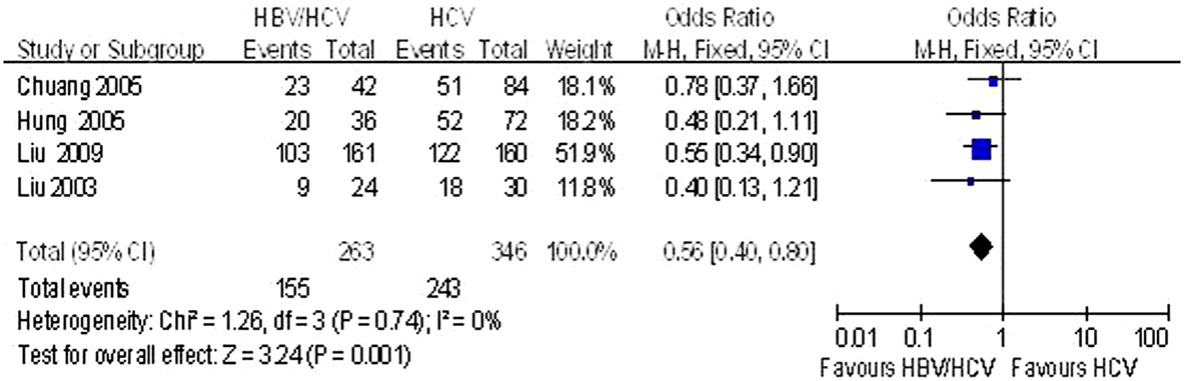
Comparison of the ALT normalization rates achieved at the end of follow up in HBV/HCV coinfection and HCV mono-infection patients.

**Figure 3:**
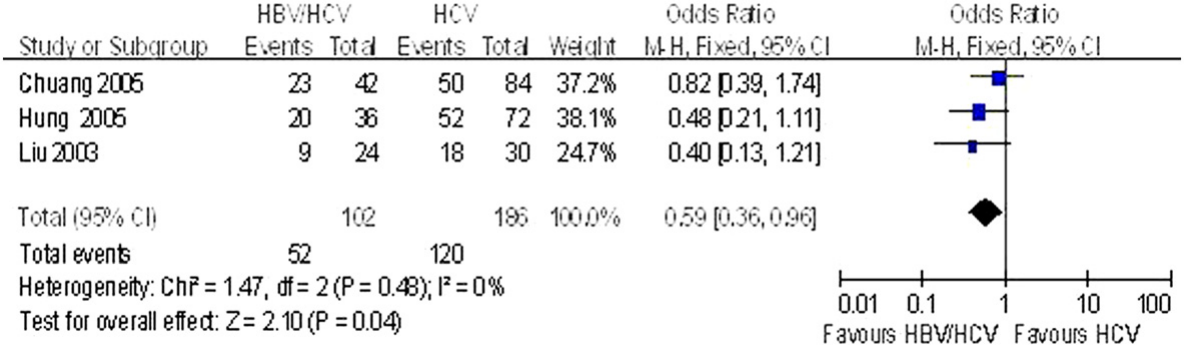
Comparison of the ALT normalization rates achieved at the end of treatment in HBV/HCV coinfection and HCV mono-infection patients.

Four of the studies [30,32,34,36] reported end of follow-up ALT normalization. Meta-analysis revealed that patients with HBV/HCV coinfection have lower serum ALT normalization than those with HCV mono-infection at the end of follow-up (*P* = 0.001; Figure 2). ALT normalization at the end of treatment in coinfection groups was also lower than in the HCV mono-infection groups (*P* = 0.04; Figure 3).

### Comparison of ETVR and SVR of HCV achieved in HBV/HCV coinfection patients and HCV mono-infection patients

The ETVR rate was similar between HBV/HCV coinfection groups and HCV mono-infection groups at the end of treatment (85.98% vs. 86.36%, *P* = 0.96). Morover, there was no significant difference in the SVR rate between the two groups at the end of follow-up (68.05% vs. 70.41%, *P* = 0.38). Detailed information can be found in Table 3.

**Table 3:** Pooled effect of HBV/HCV coinfection studies with HCV mono-infection control studies

**Effects**	**HBV/HCV**			**HCV**		**Effect size**		**Heterogeneity**	
	**Studies (*n*)**	**Sample size**	**Events**	**Sample size**	**Events**	**OR(95% CI)**	***P***	***I***^***2***^	***P***
ETVR	4	271	233	308	266	1.03(0.37-2.82)	0.96	69%	0.02
SVR	5	313	213	392	276	0.87(0.62-1.21)	0.38	0%	0.61
HCV relapse	4	270	49	308	42	1.55(0.98-2.47)	0.06	47%	0.13
HCV genotype 1 relapse	2	127	27	135	13	2.4(1.17-4.91)	0.19	74%	0.05
HCV non-genotype 1 relapse	2	84	7	71	7	0.9(0.3-2.71)	0.85	0%	0.4o

*OR* odds ratio.

### Comparison of the relapse of HCV between HBV/HCV coinfection and HCV mono-infection

The relapse of HCV rate was similar between HBV/HCV coinfection groups and HCV mono-infection groups at the end of follow-up (18.15% vs. 13.64%, *P* = 0.06). The relapse of rate of HCV was also analyzed according to HCV genotype 1 and HCV non-genotype 1 (Table 3). The relapse of rates in both HCV genotype groups were similar between the HBV/HCV coinfection groups and HCV mono-infection groups at the end of follow-up (HCV genotype 1: 21.26% vs. 9.62%, *P* = 0.19; HCV non-genotype 1: 8.33% vs. 9.86%, *P* = 0.85) (Table 3).

### Viral interaction of HBV and HCV in HCV/HBV coinfection patients

**Figure 4:**
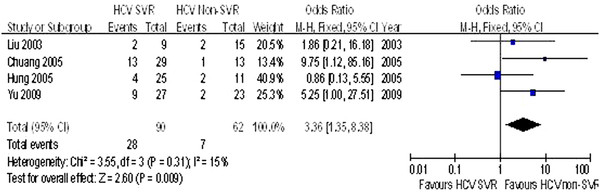
Comparison of the HBVDNA resurgence HCV SVR groups and HCV non-SVR groups in HBV/HCV coninfection patients.

All five [30,32,34-36] of the studies reported data for the HBV DNA level at baseline and at the end of follow-up in HBV/HCV coinfection patients. In addition, data for the HBV DNA resurgence in patients with and without HCV SVR were reported (Table 4). The rate of HBV DNA resurgence in HBV/HCV coinfection patients with HCV SVR was significantly higher than in those without HCV SVR (OR = 3.36, 95% CI: 1.35–8.38, *P* = 0.009, Figure 4).

**Table 4:** Viral interaction of HBV and HCV in HCV/HBV-coinfected patients

**Study**	**Geographic locale**	**Sample size (*n*)**	**Baseline HBV DNA (*n*)**		**End of follow-up (*n*)**					
					**HCV SVR**	**Non- HCV SVR**	**Baseline HBV DNA positive**	**HBV DNA resurgence**		
			**Positive**	**Negative**			**HBV VR**	**Overall**	**HCV SVR**	**Non- HCV SVR**
Yu et al. [35]	China	50	4	46	27	23	4	11	9	2
Liu et al. [32]	Taiwan	161	76	85	123	38	38	17	NA	NA
Chuang et al. [34]	Taiwan	42	16	26	29	13	5	14	13	1
Hung et al. [30]	Taiwan	36	18	18	25	11	3	6	4	2
Liu et al. [36]	Taiwan	24	20	4	9	15	6	4	2	2

*NA* not available, *VR* virological response (undetectable HBV DNA).

## Discussion

Several reports have revealed that HBV and HCV are capable of suppressing replication of each other in coinfection conditions. Dominant roles for both HCV and HBV have been suggested by some investigators [10,12,13]. However, our meta-analysis indicates that HBV DNA was not always detectable in patients with HBV/HCV coinfection, and that the rate of HBV DNA resurgence in HBV/HCV coinfection patients with HCV SVR was significantly higher than in those without HCV SVR (31.11% vs. 11.29%, *P* = 0.009). These findings are consistent with the idea that patients with HBV/HCV coinfection show a large spectrum of virological profiles. It is possible that dominance of HBV or HCV may alternate at different periods during the infection. Thus, we speculate that one of the viruses in patients with HBV/HCV coinfection is capable of exerting its particular pathogenic role and masking or silencing that of the other virus. Moreover, once the dominant virus becomes suppressed by treatment, the other may have a tendency towards reactivation. This type of robust activity elicited by one virus can explain the increased severity of disease observed in patients with HBV/HCV coinfection, as opposed to that in patients with mono-infection of either of the two viruses.

The most commonly used clinical therapy to treat either HBV or HCV is interferon, usually administered as a subcutaneous injection. This drug was developed based upon the normal host antiviral and immunomodulatory actions that target invading viral pathogens for destruction and clearance. Previous studies have shown that patients with HCV mono-infection respond remarkably better to combination therapy (interferon supplemented with the nucleoside analogue ribavirin) than to interferon monotherapy (SVR of 43% vs. 10%) [27,29,37,38]. Moreover, patients with CHB treated with conventional interferon achieved an SVR of 35% [28], and use of PEG-IFN achieves an even higher SVR in patients with CHB and CHC [39,40]. To date, interferon has been the most studied pharmacologic agent for treatment of HBV/HCV coinfected patients, because of its proven activity against both viruses. The efficacy of combination treatment with interferon plus ribavirin in HBV/HCV coinfection patients has been assessed in various studies [41,42], but very few studies to date have comparatively analyzed HBV/HCV coinfection and HCV mono-infection, and the conclusions have been largely discordant [30-32]. Liu et al. [35] reported that the efficacy of combination therapy with PEG-IFN and ribavirin was similar between HBV/HCV coinfected and HCV monoinfected patients. However, another study [34] showed that the HBV/HCV coinfected patients had higher ETVR and relapse rates than monoinfected patients.

Our meta-analysis confirmed that the combination treatment approach, with either conventional interferon or PEG-IFN plus ribavirin, achieved comparable ETVR and SVR in patients with HBV/HCV coinfection and those with HCV mono-infection. Our analysis also showed that patients with HBV/HCV coinfection were at similar risk of HCV relapse to those with HCV mono-infection, regardless of the presence of HCV genotype 1 or HCV non-genotype 1. However, combination treatment achieved higher ALT normalization in HCV mono-infection patients than in those with HBV/HCV coinfection by the ends of both treatment and follow-up. According to the finding that eradication of one hepatitis virus in patients with HBV/HCV coinfection may lead to increased titer of the other, we speculate that the resurgence of HBV might account for the persistent hepatitis activity after the clearance of HCV. Therefore, examination of HBV antigens and HCV replication in the liver compartment is required to confirm this hypothesis.

These findings may provide insights into why the ALT normalization rate in patients with HBV/HCV coinfection was lower than that in patients with HCV mono-infection. Yet undefined viral interactions and their impacts on treatment efficacy (for example, producing similar ETVR and SVR but lower ALT normalization) may explain the suppressive effect of HBV on HCV that has been observed in HBV/HCV coinfected patients. Once the dominant HBV becomes suppressed by IFN-based therapy, coinfected patients may experience HCV reactivation and manifest HCV-specific symptoms [21]. Therefore, clinicians should exercise caution when treating coinfected patients with combination therapy (the combination of PEG-IFN and ribavirin being the preferred strategy), and perform careful follow-up with systematic supervision. Larger scale studies should be carried out to determine whether prolonging the course of antiviral treatment in patients with HBV/HCV coinfection will increase the risk of ALT normalization.

There are several limitations to our meta-analysis that should be considered prior to generalization of our findings. First, these five studies were composed exclusively of individuals of Asian descent. Second, conclusions were made based upon sub-analyses using calculated *p*-heterogeneity values. Third, the studies were not identical in the administered doses of interferon and ribavirin, types of interferon administered, or course of treatment; these differences in study design may explain the statistical heterogeneity. Fourth, the data of HCV relapse and ALT normalization in genotype 1 and non-genotype 1 infected patients were unavailable in some studies, which may have affected the accuracy of this meta-analysis.

In conclusion, the results of our meta-analysis demonstrate that combination treatment with interferon plus ribavirin achieves similar ETVR and SVR in HBV/HCV coinfection patients and HCV mono-infection patients. However, HBV/HCV coinfection patients achieve significantly lower ALT normalization and are at significantly higher risk of relapse. The combination of PEG-IFN and ribavirin is more effective than that of conventional interferon and ribavirin for both coinfection and mono-infection.

## Abbreviations

HBV: Hepatitis B Virus; HCC: Hepatocellular Carcinoma; CHB: Chronic Hepatitis B; CHC: Chronic Hepatitis C; HBsAg: Hepatitis B Surface Antigen; ALT: Alanine Aminotransferase; ETVR: End-of-Treatment Virological Response; SVR: Sustained Virological Response; HIV: Human Immunodeficiency Virus; RR: Relative Risk; CI: Confidence Interval; PEG-IFN: Pegylated-Interferon.

## Competing interests

The funding source had no influence on study design, on the collection, analysis, and interpretation of the data, on the writing of the manuscript, or on the decision to submit this manuscript for publication. The contents are solely the responsibility of the authors and do not necessarily represent the views of the funding source.

## Authors’ contributions

RH and HP conceived the study, provided funding support, and revised the manuscript critically for intellectual content. LJY made substantial contributions to study design and data acquisition, analysis and interpretation. SYJ, TSW, HHD, ZQ, WJ, ZDZ and ZZ participated in the design of the study and data acquisition, analysis and interpretation. All authors read and approved the final manuscript.

## Supplementary Material

Additional file 1 PRISMA 2009 Checklist. Click here for file
